# Post-immunization leucocytosis and its implications for the management of febrile infants

**DOI:** 10.1016/j.vaccine.2018.03.026

**Published:** 2018-05-11

**Authors:** Sarah Prentice, Zephyrian Kamushaaga, Stephen B. Nash, Alison M. Elliott, Hazel M. Dockrell, Stephen Cose

**Affiliations:** a Department of Clinical Research, London School of Hygiene and Tropical Medicine, Keppel Street, London WC1E 7HT, United Kingdom; b MRC/UVRI Uganda Research Unit, 51-59 Nakiwogo Road, Entebbe, PO Box 49, Uganda; c Department of Infectious Disease Epidemiology, London School of Hygiene and Tropical Medicine, Keppel Street, London WC1E 7HT, United Kingdom; d Department of Immunology and Infection, London School of Hygiene and Tropical Medicine, Keppel Street, London WC1E 7HT, United Kingdom

**Keywords:** Immunization, Leucocytosis, Fever, Clinical management, Infant

## Abstract

**Aims:**

Clinical guidelines for management of infants with fever but no evident focus of infection recommend that those aged 1–3 months with a white cell count >15 × 10^9^/l have a full septic screen and be admitted for parenteral antibiotics. However, there is limited information about leucocyte changes following routine immunization, a common cause of fever. We investigated white cell counts shortly after routine immunization in Ugandan infants under 3 months of age.

**Methods:**

White cell counts were measured in 212 healthy infants following routine immunizations (DTwP-HepB-Hib, oral polio and pneumococcal conjugate 7 vaccines) received prior to 3 months of age.

**Results:**

Mean leucocyte counts increased from 9.03 × 10^9^/l (95% confidence interval 8.59–9.47 × 10^9^/l) pre-immunizations to 16.46 × 10^9^/l (15.4–17.52 × 10^9^/l) at one-day post-immunizations at 6 weeks of age, and 15.21 × 10^9^/l (14.07–16.36 × 10^9^/l) at one-day post-immunizations at 10 weeks of age. The leucocytosis was primarily a neutrophilia, with neutrophil percentages one-day post-immunization of 49% at 6 weeks of age and 46% at 10 weeks of age. White cell parameters returned to baseline by two-days post-immunization. No participant received antibiotics when presenting with isolated fever post-immunization and all remained well at follow-up.

**Conclusions:**

In our study almost half the children <3 months old presenting with fever but no evident focus of infection at one-day post-immunization met commonly used criteria for full septic screen and admission for parenteral antibiotics, despite having no serious bacterial infection. These findings add to the growing body of literature that questions the utility of white blood cell measurement in identification of young infants at risk of serious bacterial infections, particularly in the context of recent immunizations, and suggest that further exploration of the effect of different immunization regimes on white cell counts is needed.

This observational work was nested within a clinical trial, registration number ISRCTN59683017.

## 1 Introduction

Fever is one of the most common reasons for presentation of children to medical professionals [1]. Children presenting with no obvious focus for their infection can pose a diagnostic challenge to clinicians. Algorithms exist to assist in the identification of children who would benefit from investigation and admission to hospital for treatment. These guidelines are particularly stringent for febrile infants less than 3 months old, due to the increased risk of occult serious bacterial infections [2]. Guidelines used in the UK [3], and in adapted forms worldwide, advise that a full blood count and partial septic screen should be performed on any infant presenting with a fever >38 °C without focus when less than 3 months of age, even if otherwise well-looking and regardless of recent immunization history. Infants who have a white cell count of >15 × 10^9^/l are then admitted to hospital for a full septic screen, including lumbar puncture, and parenteral antibiotics whilst culture results are pending (usually a minimum of 48 h).

Infants worldwide commonly receive a number of vaccinations in the first few months of life, generally with multiple antigens administered on one day [4]. These vaccines are highly immunostimulatory and the occurrence of fever >38 °C following routine vaccinations is well recognised. However, the effect on white cell counts of the co-administration of multiple vaccine antigens, such as those received during primary immunizations, is unknown. Studies conducted in the 1980s in Finland and the USA in a small number of older infants, showed an increase in white cell counts post administration of the combined Diptheria-Tetanus-whole cell Pertussis (DTwP) vaccination [5]. However, few similar studies have been published looking at younger infants and using the enhanced combination of vaccine antigens currently in use.

Lack of knowledge regarding alterations to white cell count levels following routine immunization could severely impede clinical decision making during the assessment of a feverish child. This may have negative consequences for the child due to unnecessary invasive investigations and antibiotic administration. This study investigated alterations to white cell counts during the period immediately following routine immunization, in the first 3 months of life.

## 2 Methods

Post-immunization blood samples were collected from 212 Ugandan infants as part of a randomised controlled trial investigating the impact of BCG vaccination on the innate immune system (described elsewhere [6]). In brief, infants were randomised to receive BCG either at birth or at 6 weeks of age. All other routine immunizations were given as per Ugandan national guidelines: oral polio vaccine (OPV) at birth and pentavalent vaccine (diptheria-tetanus-whole-cell pertussis/*Haemophilus influenzae* B/Hepatitis B), OPV and pneumococcal vaccine (PCV10) at 6 weeks, 10 weeks and 14 weeks of age (hereafter referred to as ‘primary immunization’). Infants were then randomly assigned to have venous blood samples taken on two out of four possible time points: (1) 5 days of age, (2) 6 weeks of age, 1 day following immunization, (3) 6 weeks of age, 5 days following routine immunization and (4) 10 weeks of age, 1 day following routine immunization. In practice, blood samples were taken at a range of times post-routine immunization, due to delayed attendance at clinic for some participants. Infants with blood samples taken more than 15 days following immunization were excluded from analysis (n = 1). BCG vaccination in the delayed group was given after blood sample 2 but prior to blood sample 3. However, upon analysis, no significant impact of the different BCG schedules on white blood cell count was shown and data were analysed together.

Anthropometry, vital sign measurement and clinician review of participants occurred at each appointment. Temperatures were measured using a digital axillary thermometer, following current best practice recommendations. Active follow-up of participants occurred for the duration of the trial with open access to clinician review and treatment, as well as weekly telephone follow-up, to confirm health status.

Full blood counts were obtained using the automated Coulter AcT 5diff CP (Beckman-Coulter, California, USA), from 0.5 ml of venous blood drawn from the dorsum of the hands or feet into an EDTA containing microtainer (Becton-Dickson).

Data were analysed using STATA version 14.1 (StataCorp, Texas, USA) and graphs produced using Prism 6 (GraphPad Software, Inc. California, USA). Results were normally distributed so means with 95% confidence intervals are reported, with Student’s *t*-test used for comparison of means pre- and post-immunization. Changes in mean values over time were analysed using a random effects model to account for repeated measurements and including both linear and quadratic terms for time to allow for a non-linear relationship.

Ethical approval for the trial was obtained from the Uganda Virus Research Institute Research and Ethics Committee (Ref: GC/127/13/11/432), the Uganda National Council for Science and Technology (Ref: HS 1524), The Office of the President of Uganda and the London School of Hygiene & Tropical Medicine (Ref: 6545). The study was conducted according to the principles of the Declaration of Helsinki. Written, informed consent of mothers was obtained by trained study nurses prior to any procedures.

## 3 Results

Two hundred and twelve infants provided blood samples for this study, 49% of them male. The background of the population was East African, primarily of the Buganda tribe and participants came from a mixture of urban, semi-urban and rural fishing communities. No participant was severely malnourished at the time of blood sample collection.

**Fig. 1:**
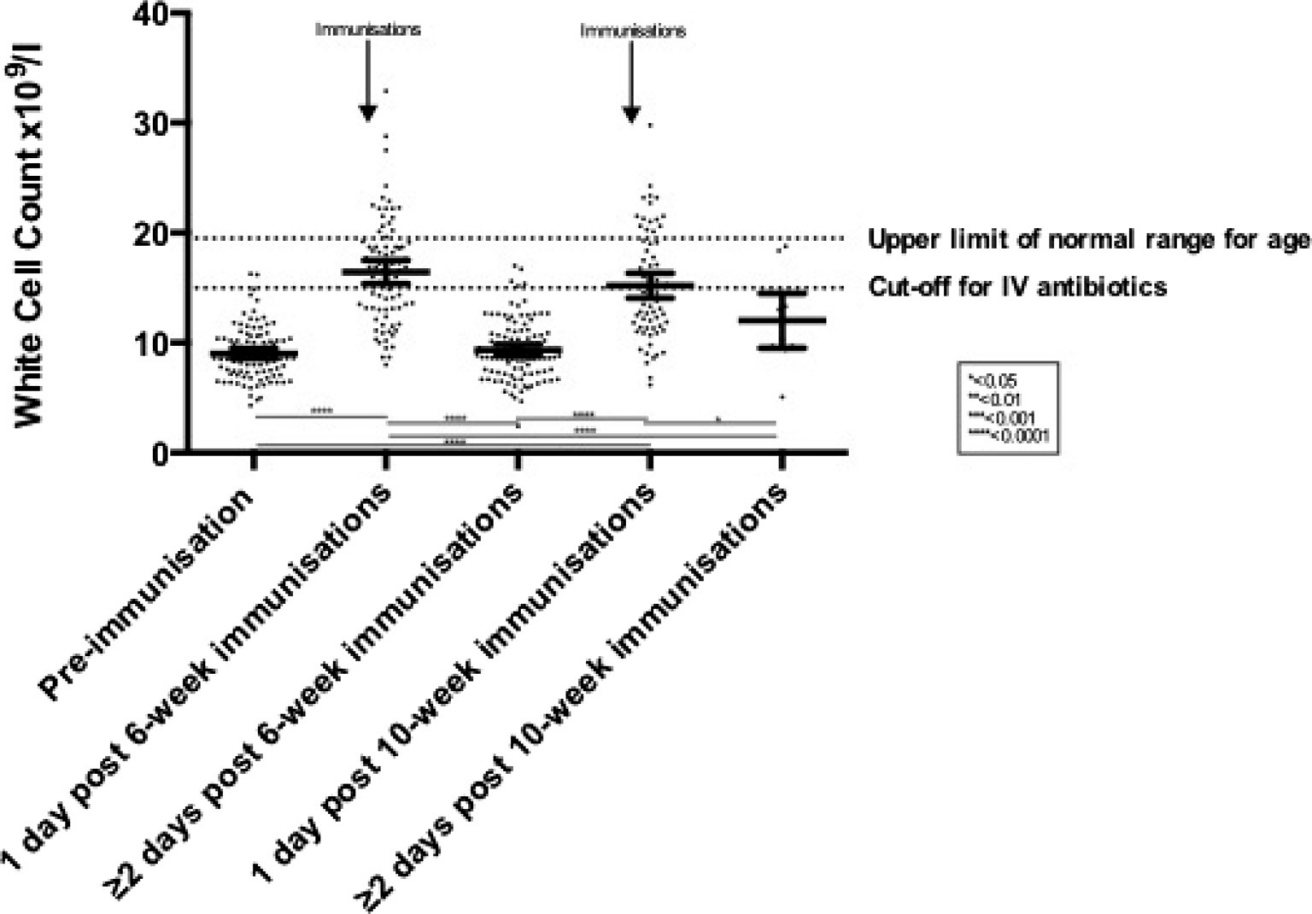
Total white cell counts by immunization status. Individual data points are represented by dots. Error bars display the 95% confidence interval.

Average white cell counts were significantly increased at one-day post receipt of primary immunizations at both 6 weeks of age (16.46 × 10^9^/l (95% confidence interval 15.40–17.52 × 10^9^/l) and 10 weeks of age (15.21 × 10^9^/l (14.07–16.36 × 10^9^/l)), compared to pre-immunization values (9.03 × 10^9^/l (8.59–9.47 × 10^9^/l), p-values for difference with post-immunization levels <0.0001, see Table 1 and Fig. 1).

**Table 1:** Blood count parameters in relation to primary immunizations CI: confidence interval.

	Pre-immunizations	6 weeks of age		10 weeks of age	
	Mean age 10 days (range 2–19 days)	1-day post-primary immunizations	≥2-days post-primary immunization (mean 5.7 days)	1-day post-primary immunization	≥2-days post primary immunization (mean 3.4 days)
	n = 106	n = 81	n = 111	n = 70	n = 12
Total White Cell Count × 10^9^/l (95% CI)	9.03 (8.59–9.47)	16.46 (15.40–17.52)	9.34 (8.84–9.84)	15.21 (14.07–16.36)	12.02 (9.51–14.52)

Subset Counts × 10^9^/l (95% CI)					
Neutrophils	2.65 (2.42–2.88)	8.58 (7.64–9.52)	2.21 (2.02–2.41)	7.00 (6.36–7.64)	3.07 (1.94–4.20)
Lymphocytes	4.64 (4.40–4.87)	6.15 (5.78–6.51)	5.81 (5.48–6.14)	6.24 (5.73–6.76)	7.22 (5.80–8.64)
Monocytes	1.13 (1.04–1.22)	1.70 (1.55–1.85)	0.92 (0.85–0.99)	1.43 (1.27–1.58)	1.06 (0.74–1.38)
Eosinophils	0.35 (0.31–0.38)	0.14 (0.12–0.16)	0.27 (0.23–0.30)	0.33 (0.28–0.38)	0.46 (0.26–0.66)
Basophils	0.26 (0.22–0.31)	0.26 (0.23–0.29)	0.14 (0.12–0.15)	0.22 (0.19–0.25)	0.21 (0.15–0.27)

Percentage (95% CI)					
Neutrophils	28.6 (27.08–30.12)	48.89 (46.98–50.80)	23.54 (22.12–24.97)	45.87 (43.93–47.81)	24.96 (19.54–30.37)
Lymphocytes	52.19 (50.36–54.03)	38.42 (36.58–40.27)	62.28 (60.68–63.88)	41.41 (39.42–43.39)	61.05 (54.72–67.37)
Monocytes	12.44 (11.79–13.09)	10.30 (9.77–10.82)	9.86 (9.33–10.38)	9.22 (8.71–9.73)	8.40 (7.08–9.72)
Eosinophils	3.96 (3.52–4.39)	0.88 (0.78–0.97)	2.92 (2.52–3.32)	2.17 (1.90–2.44)	3.94 (2.35–5.53)
Basophils	2.80 (2.38–3.21)	1.48 (1.39–1.58)	1.41 (1.33–1.49)	1.32 (1.22–1.43)	1.67 (1.35–1.99)

Haemoglobin g/dl (95% CI)	15.98 (15.57–16.38)	10.68 (10.39–10.96)	11.09 (10.87–11.31)	10.30 (10.07–10.54)	10.79 (10.24–11.34)

Platelet counts × 10^9^/l (95% CI)	362.10 (337.08–387.11)	524.52 (493.86–555.17)	575.62 (547.11–604.13)	443.26 (416.87–469.64)	520.42 (455.12–585.71)

**Fig. 2:**
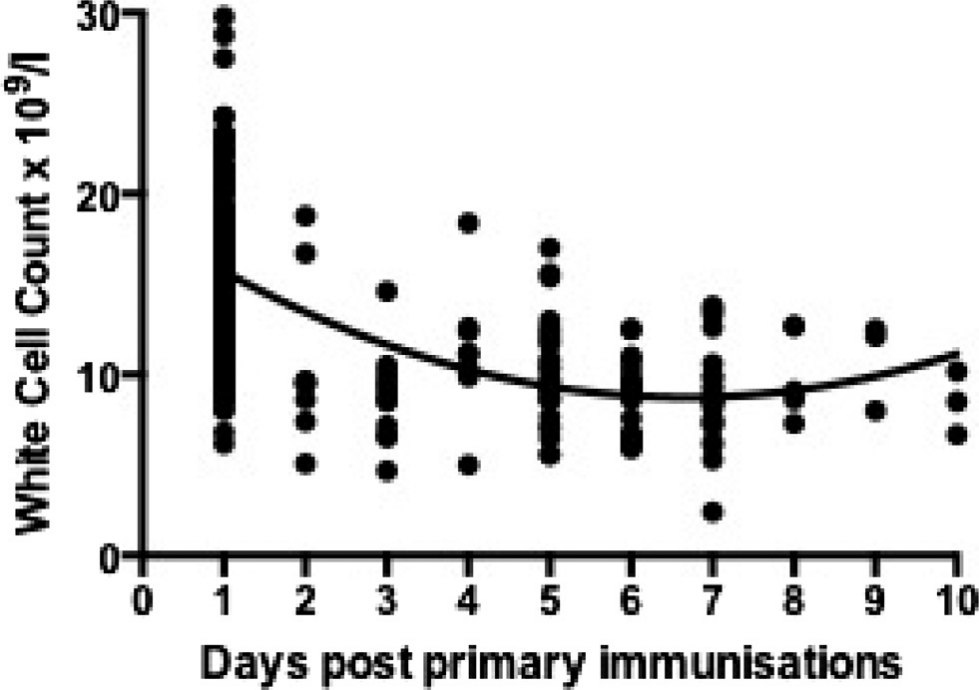
White cell count by time post-immunizations. Individual data points are represented by dots. The line represents results of the random effects regression model.

This rise in mean total leucocytes was short-lived, returning to levels not significantly different from baseline by two days post-immunization, but continuing to decline up to six-days post-immunization (p < 0.0001) (Fig. 2). Although mean white cell counts at one day post-immunization fell within the normal range expected for age (5.0–19.5 × 10^9^/l) [7], there was a wide range of values (8.00–32.90 × 10^9^/l at one-day post 6-week immunization and 6.20–29.80 × 10^9^/l at one day post 10-week immunization). At both time-points an average of 22% of white cell counts measured fell outside of the normal range for age. At one day post-immunization, on average 53% of measured white cell counts were above the 15 × 10^9^/l cut-off for further intervention when managing a febrile child <3 months old (Fig. 1).

**Fig. 3:**
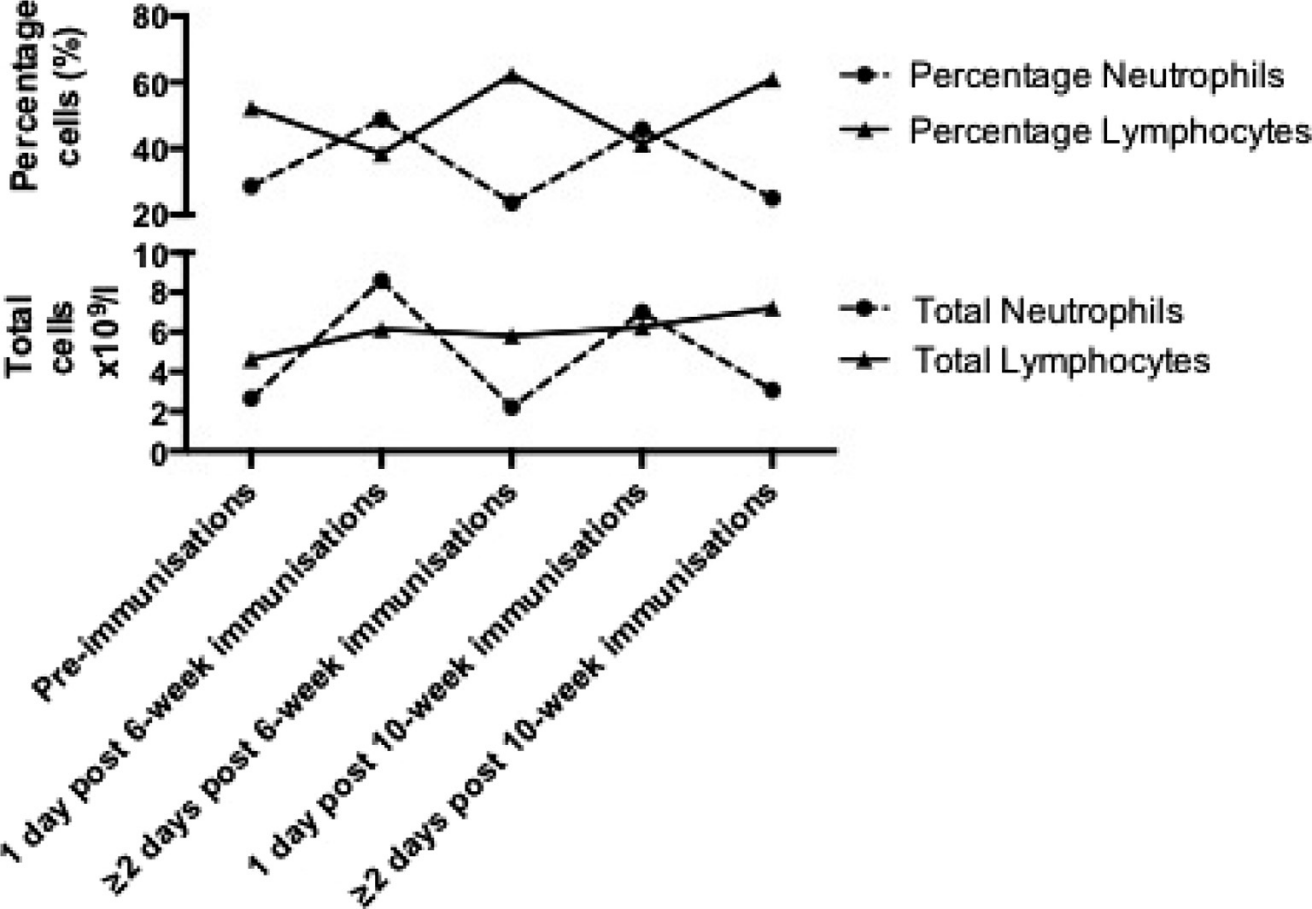
Total and percentage neutrophils and leucocytes by immunization status.

The leucocytosis observed at one-day post immunization was primarily a neutrophilia (Table 1 and Fig. 3). Little change occurred to total lymphocyte levels, other than an expected increase with age (see Fig. 3). As a result at one-day post-primary immunization, the percentage of the white cell count made up by lymphocytes dropped as the percentage accounted for by neutrophils increased (Table 1 and Fig. 3). The average percentage of neutrophils was above the normal range for age (up to 32% neutrophils [7]) at one-day post-primary immunization at both 6 weeks of age (49%) and 10 weeks of age (46%). Total monocyte and basophil levels mimicked changes to neutrophils post-immunization, though to a much smaller extent (Table 1). The reverse occurred with eosinophils, with total eosinophils dropping at 1-day post-immunization and rising by day 2. The changes to monocyte, basophil and eosinophil count were only significant at the 6-week time-point. There was little change to the percentage of monocytes, eosinophils and basophils by immunization status.

**Fig. 4:**
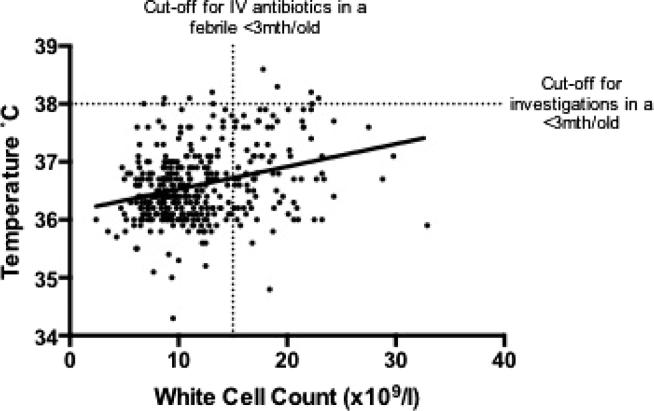
Axillary temperature of children in relation to their white cell count. Individual data points are represented by dots. The line represents results of the linear regression model.

Linear regression analysis provided good evidence (p < 0.0001) of a weak, positive association of temperature and white cell counts, with each one degree Celsius increase in temperature associated with a 0.04 × 10^9^/l increase in white cell count (Fig. 4). Of all children studied that presented with a fever >38 °C when the blood sample was taken, 5 out of 11 (45%) had a white cell count above the currently recommended threshold for further investigation and inpatient management with IV antibiotics. A further 17 mothers reported that their children had been pyrexial prior to presentation. Of these, 3 (18%) had white cell counts above 15 × 10^9^/l. All children presenting with either fever >38 °C or with parental report of fever were clinically assessed as being well and treated conservatively as outpatients without antibiotics. All remained well at follow-up and no cases of serious bacterial infection occurred. Eighty-five infants had white cell counts >15 × 10^9^/l, but were afebrile, with 28 of these having white cell counts above the normal reference range for age.

These data provided no evidence that either BCG immunization status or gender had any impact on results. There was also no evidence of a difference in mean haemoglobin and platelet counts comparing pre- and post-immunization levels, other than an expected decrease in haemoglobin with increasing infant age (see Table 1).

## 4 Discussion

This study shows a rapid and large increase in white cells, primarily neutrophils, occurring in infants <3 months old immediately following primary immunizations. This increase is above current guideline thresholds for further investigation and treatment in nearly half of febrile infants studied and above the normal white cell count range for age in more than a quarter of infants studied. These infants all remained well during the post-immunization period, in the absence of intervention, and mean white cell counts returned to baseline by two-days post-immunization. These infants therefore represent a group that may cause diagnostic confusion and undergo unnecessary investigations and interventions if they present to a clinician febrile, or if they have a blood test taken for an unrelated condition, at one-day post-immunization. The development of new post-immunization reference ranges could help to mitigate this. In the absence of other data for our population, our study would suggest a reference range of total leucocytes: 7.76–27.25 × 10^9^/l, percentage neutrophils: 29%-65% (2.5–97.5th centiles [8]) as appropriate for infants less than 3 months old, one-day following routine immunizations.

This study’s strengths lie in its comparatively large study numbers, giving robust results, and the presence of blood samples from a variety of time-points post-primary immunizations, allowing the timing of changes in white cell counts post-primary immunizations to be investigated. The close follow-up of participants during the post-immunization period provides reassuring evidence that children with fevers and high white cell counts immediately following immunization can remain well without further intervention.

The study has a number of limitations. Firstly, it is a secondary analysis conducted as part of a larger randomised controlled trial that was not specifically designed to look at white cell counts in post-immunization pyrexia. As a result, the number of febrile infants in the study was limited. However, the correlation between temperature and white cell count seen in our study suggests that these results can be extrapolated to febrile infants more generally, with higher white cell counts expected in those infants that have post-immunization pyrexia. Supporting this theory, a study investigating serious bacterial infections in recently immunized infants in the USA similarly showed an increase in white cell counts in recently immunized febrile infants with no serious bacterial infection [9]. The finding of increased white cell counts in afebrile infants post-immunization is also important, as nearly a quarter of cases in our study fell outside the normal range. These cases might cause diagnostic confusion if blood is sampled following immunizations for another reason.

Another limitation of this study is that the time course of changes to white cell counts post-immunizations could be examined only because some participants did not attend their per-protocol appointments at the correct time (24 h or 5 days post-immunizations). It may be argued that these participants represent a different sub-set of the population, for instance infants who had fewer post-immunization symptoms, and may therefore have falsely lower white cell counts than the population as a whole. However, the time-course of white cell count changes followed a logical pattern with average levels declining until day 7 post-immunization (which encompassed the per-protocol appointment day 5 post-immunizations) and mirrored the time-course of changes to IL-6 and CRP that have been shown post-DTwP immunization in another study [10]. Also, the timing of blood samples used to obtain pre-immunization average white cell counts was at an average of 10 days of post-natal age, rather than immediately prior to the receipt of primary immunizations, due to the design requirements of the parent trial. This comparison was deemed to be acceptable, however, as white cell counts are known to be high at birth, falling to adult levels by approximately 2 weeks of age [11]. Samples taken at an average of 10 days of post-natal age would therefore be more likely to under-estimate the degree of change in white cell counts following primary immunizations, rather than falsely over-estimate it.

The generalizability of this study’s findings may be limited due to its restricted study population and the choice of vaccine combination used for primary immunization. As the study was conducted in Uganda, the ethnicity of infants was solely black African. White cell counts in black Africans, however, tend to be lower than in other ethnic groups [12], [13], [14]. It is therefore possible that white cell count changes post-primary immunization would be at least as marked, if not more, in other populations. Previous studies conducted in white European and mixed American populations have also shown white cell count increases at one-day post-immunization [5], [9], though to a lesser extent than with the combination of vaccines used in this study.

The combination of vaccines used as primary immunizations is not the same throughout the world and this may limit the global applicability of these findings. Most primary immunization regimes include components against diptheria, tetanus, pertussis, *Haemophilus influenzae* type B and pneumococcus (as were included in this study) [4]. However, the use of oral polio vaccine has been replaced in high-income countries with an inactivated vaccine [15], and immunization against hepatitis B is often only given to those deemed at high risk. Additional vaccines, not used in this study, such as meningococcal and rotavirus vaccine are also commonplace in many other areas of the world. The differences in vaccine components used may cause variations in the degree of post-immunization leukocytosis. Of these, the replacement of whole cell pertussis (used in this study and in many low income countries as part of the 5-in-1 vaccine) with acellular pertussis (used in many European and North American countries) may have the most impact on post-immunization leukocytosis [10], [16], though a study into serious bacterial infections in the context of post-immunization pyrexia used DTaP and also revealed a raised white cell count post-immunizations [9]. A previous study conducted in Gambian neonates [17] showed no increase in white cell counts following oral polio and hepatitis B vaccination (as well as BCG), suggesting that it was not these components of primary immunizations that were responsible for post-immunization leukocytosis (unpublished findings), and thus the discontinuation of their use in high-income countries might not affect results. The addition of further antigens/adjuvants/vaccines to the basic vaccine combination used in this study may be hypothesized to further increase immunostimulation and white cell counts, rather than diminish them. Thus, the recommendations of this study may be a conservative estimation of changes occurring in other areas of the world. However, further studies in different settings would be necessary for the development of a robust global reference range for post-immunization white cell counts. The timing of primary immunizations also varies globally, which may affect a child’s post-immunization white cell count response. However, this study showed similar increases in white cell counts at 6 weeks and 10 weeks of age, suggesting that small variations in immunization timing are unlikely to affect overall responses.

This study adds to the current debate regarding the utility of white cell counts in the assessment of children who present febrile with no clear focus for infection. Since the introduction of immunizations against *Streptococcus pneumonia* and *Haemophilus influenza*, the incidence of serious bacterial infections in young febrile infants has reduced [18]. Several studies have subsequently found that a cut-off of 15 × 10^9^/l white cells is neither sensitive nor specific for the identification of serious bacterial infections in febrile children [19], [20], [21], [22], [23], [24], [25], [26], [27]. Newer proposed algorithms for assessment of fever with no focus have tended to relegate this parameter in favour of other markers of infection, such as CRP and procalcitonin [28], [29]. However, these new algorithms have not been widely adopted at present. We suggest that, particularly in the context of immunization within the previous 24 h, white cell count should not be used as a discriminatory factor when deciding whether to admit and treat children under the age of 3 months old who present with fever and no source of infection. If the use of white cell counts is continued, we suggest that policymakers consider introducing either a higher white cell count threshold for further investigation and management in an otherwise well child <3 months old presenting one day post-immunizations, or a provision for a 24-h observation period with repeat white cell count, into the current guidelines for the treatment of febrile infants. This would reduce harm to patients by avoiding unnecessary invasive procedures and antibiotics, and reduce the burden on paediatric healthcare systems.

## Declaration of competing interests

All authors have completed the Unified Competing Interest form at www.icmje.org/coi_disclosure.pdf (available on request from the corresponding author) and declare that SP, ZK, HMD, AME and SC have no financial or non-financial interests that may be relevant to the submitted work.

## Details of contributors

SP was responsible for the study design, conduct, data collection, data analysis and manuscript preparation. ZK performed the white cell counts using the automated Coulter Counter. SN provided statistical support. HMD, AME and SC provided advice and support for all aspects of the above work including manuscript preparation. All authors read and approved the final manuscript.

This study was funded by a Wellcome Trust Clinical Fellowship, Grant No. ICTRZB84 and sponsored by the London School of Hygiene and Tropical Medicine. The study funder and sponsor had no input in the study design; in the collection, analysis, and interpretation of data; in the writing of the report; and in the decision to submit the article for publication. The researchers and funder remain independent. AME was supported by Wellcome Trust Grant No. 095778, SC by Wellcome Trust Grant No. 084344 and MRC Grant No. MR/K019708.

SP and SN had full access to the data (including statistical reports and tables) and can take responsibility for the integrity of the data and the accuracy of the data analysis.

## Transparency declaration

SP affirms that the manuscript is an honest, accurate, and transparent account of the study being reported; that no important aspects of the study have been omitted; and that any discrepancies from the study as planned and registered have been explained. Some of these results were presented at the Royal College of Paediatrics and Child Health Conference, April 2016.

## Data sharing statement

Data is available upon request from the principal author.

## References

[1] Nawar E.W., Niska R.W., Xu J. (2007). National Hospital Ambulatory Medical Care Survey: 2005 emergency department summary. Adv Data.

[2] American College of Emergency Physicians Clinical Policies C, American College of Emergency Physicians Clinical Policies Subcommittee on Pediatric F: Clinical policy for children younger than three years presenting to the emergency department with fever. Ann Emerg Med 2003;42:530–45. 10.1067/s0196-0644(03)00628-0 14520324

[3] Fever in under 5s: assessment and initial management. NICE guideline [CG160]. <https://http://www.nice.org.uk/guidance/cg160>.

[4] The Expanded Programme on Immunization. <http://www.who.int/immunization/programmes_systems/supply_chain/benefits_of_immunization/en/>.

[5] Mink C.M., Uhari M., Blumberg D.A., Knip M., Lewis K., Christenson P.D. (1990). Metabolic and hematologic effects and immune complex formation related to pertussis immunization. Pediatr Res.

[6] Prentice S., Webb E.L., Dockrell H.M., Kaleebu P., Elliott A.M., Cose S. (2015). Investigating the non-specific effects of BCG vaccination on the innate immune system in Ugandan neonates: study protocol for a randomised controlled trial. Trials.

[7] Rodak B.F., Fritsma G.A., Doig K. (2007). Haematology: clinical principles and applications.

[8] Ozarda Y. (2016). Reference intervals: current status, recent developments and future considerations. Biochem Med (Zagreb).

[9] Wolff M., Bachur R. (2009). Serious bacterial infection in recently immunized young febrile infants. Acad Emerg Med: Off J Soc Acad Emerg Med.

[10] Pourcyrous M., Korones S.B., Crouse D., Bada H.S. (1998). Interleukin-6, C-reactive protein, and abnormal cardiorespiratory responses to immunization in premature infants. Pediatrics.

[11] Zierk J., Arzideh F., Rechenauer T., Haeckel R., Rascher W., Metzler M. (2015). Age- and sex-specific dynamics in 22 hematologic and biochemical analytes from birth to adolescence. Clinical chemistry.

[12] Odutola A.A., Afolabi M.O., Jafali J., Baldeh I., Owolabi O.A., Owiafe P. (2014). Haematological and biochemical reference values of Gambian infants. Trop Med Int Health.

[13] El-Hazmi M.A., Warsy A.S. (2001). Normal reference values for hematological parameters, red cell indices, HB A2 and HB F from early childhood through adolescence in Saudis. Ann Saudi Med.

[14] Bellamy G.J., Hinchliffe R.F., Crawshaw K.C., Finn A., Bell F. (2000). Total and differential leucocyte counts in infants at 2, 5 and 13 months of age. Clin Lab Haematol.

[15] Garon J.R., Cochi S.L., Orenstein W.A. (2015). The challenge of global poliomyelitis eradication. Infect Dis Clin North Am.

[16] Cody C.L., Baraff L.J., Cherry J.D., Marcy S.M., Manclark C.R. (1981). Nature and rates of adverse reactions associated with DTP and DT immunizations in infants and children. Pediatrics.

[17] Prentice S., Jallow M.W., Prentice A.M. (2015). Group MR-IN: the effect of BCG on iron metabolism in the early neonatal period: a controlled trial in Gambian neonates. Vaccine.

[18] Rudinsky S.L., Carstairs K.L., Reardon J.M., Simon L.V., Riffenburgh R.H., Tanen D.A. (2009). Serious bacterial infections in febrile infants in the post-pneumococcal conjugate vaccine era. Acad Emerg Med: Off J Soc Acad Emerg Med.

[19] Yo C.H., Hsieh P.S., Lee S.H., Wu J.Y., Chang S.S., Tasi K.C. (2012). Comparison of the test characteristics of procalcitonin to C-reactive protein and leukocytosis for the detection of serious bacterial infections in children presenting with fever without source: a systematic review and meta-analysis. Ann Emerg Med.

[20] Galetto-Lacour A., Zamora S.A., Gervaix A. (2003). Bedside procalcitonin and C-reactive protein tests in children with fever without localizing signs of infection seen in a referral center. Pediatrics.

[21] Brown L., Shaw T., Wittlake W.A. (2005). Does leucocytosis identify bacterial infections in febrile neonates presenting to the emergency department?. Emerg Med J: EMJ.

[22] Bonsu B.K., Chb M., Harper M.B. (2003). Identifying febrile young infants with bacteremia: is the peripheral white blood cell count an accurate screen?. Ann Emerg Med.

[23] Bonsu B.K., Harper M.B. (2003). Utility of the peripheral blood white blood cell count for identifying sick young infants who need lumbar puncture. Ann Emerg Med.

[24] Thompson M., Van den Bruel A., Verbakel J., Lakhanpaul M., Haj-Hassan T., Stevens R. (2012). Systematic review and validation of prediction rules for identifying children with serious infections in emergency departments and urgent-access primary care. Health Technol Assessment.

[25] Zarkesh M., Sedaghat F., Heidarzadeh A., Tabrizi M., Bolooki-Moghadam K., Ghesmati S. (2015). Diagnostic value of IL-6, CRP, WBC, and absolute neutrophil count to predict serious bacterial infection in febrile infants. Acta Medica Iranica.

[26] Bachur R.G., Harper M.B. (2001). Predictive model for serious bacterial infections among infants younger than 3 months of age. Pediatrics.

[27] Hsiao A.L., Baker M.D. (2005). Fever in the new millennium: a review of recent studies of markers of serious bacterial infection in febrile children. Curr Opin Pediatr.

[28] Gomez B., Mintegi S., Bressan S., Da Dalt L., Gervaix A., Lacroix L. (2016). European group for validation of the step-by-step a: validation of the “Step-by-Step” approach in the management of young febrile infants. Pediatrics.

[29] Bressan S., Gomez B., Mintegi S., Da Dalt L., Blazquez D., Olaciregui I. (2012). Diagnostic performance of the lab-score in predicting severe and invasive bacterial infections in well-appearing young febrile infants. Pediatr Infect Dis J.

